# Standard reference and percentiles of maximum respiratory pressures values of healthy children aged 7–10 years

**DOI:** 10.1590/1984-0462/2022/40/2021007IN

**Published:** 2022-05-27

**Authors:** Camila Isabel Santos Schivinski, Renata Maba Gonçalves Wamosy, Paloma Lopes Francisco Parazzi, André Moreno Morcillo

**Affiliations:** a Universidade do Estado de Santa Catarina, Florianópolis, SC, Brazil.; b Universidade Estadual de Campinas, Campinas, SP, Brazil.

**Keywords:** Respiratory muscles, Children, Muscle strength, Músculos respiratórios, Criança, Força muscular

## Abstract

**Objective:**

This study aims to present standard reference for values of maximum respiratory pressures of healthy schoolchildren, according to gender.

**Methods:**

This is a cross-sectional study involving healthy children aged 7–10 years. Data of body mass and height were evaluated to calculate body mass index (BMI). In addition, forced expiratory volume in 1 second (FEV_1_) and maximal expiratory pressure values were evaluated according to the American Thoracic Society. The maximal inspiratory pressure (MIP) and data of maximal expiratory pressure (MEP) obtained in the study showed normal distribution and curves were built by the Lambda–Mu–Sigma (LMS) method, as well as the values of MIP and MEP percentiles 3, 10, 25, 50, 75, 90, and 97 for each gender.

**Results:**

MIP and MEP data were collected from 399 schoolchildren. All schoolchildren in the sample had adequate FEV_1_ and BMI. The study showed an increase in respiratory pressure values with age progression. The MIP and MEP values of girls were of 53.4±11.0 and 61.8±12.5cmH_2_O, respectively, and those of boys were 59.9±13.6 and 69.6±15.7cmH_2_O, respectively.

**Conclusions:**

Normal curves and percentiles were developed for MIP and MEP values of healthy schoolchildren. The extreme percentiles (3rd and 97th) were determined, and a specific graph was elaborated for each group. These graphs may help clinical follow-up and therapeutic monitoring of different pediatric populations.

## Resumo

**Objetivo:**

Apresentar distribuição normal para os valores das pressões respiratórias máximas de escolares saudáveis, de acordo com o gênero.

**Métodos:**

Estudo transversal envolvendo crianças saudáveis de 7 a 10 anos. Foram avaliados os dados de massa corporal e estatura, para o cálculo do índice de massa corporal (IMC). Os valores de volume expiratório forçado no primeiro segundo (VEF_1_) e pressão expiratória máxima foram avaliados de acordo com a American Thoracic Society. Os dados de pressão inspiratória máxima (PI_máx_) e pressão expiratória máxima (PE_máx_) obtidos no estudo deram origem à distribuição normal, cuja construção foi elaborada pelo método the Lambda-Mu-Sigma (LMS), bem como os percentis 3, 10, 25, 50, 75, 90 e 97 de PI_máx_ e PI_máx_ de cada sexo.

**Resultados:**

Os dados de PI_máx_ e PI_máx_ foram coletados de 399 escolares. Todos os escolares da amostra apresentavam VEF_1_ e índice de massa corporal adequados. O estudo mostrou aumento dos valores de pressão respiratória com o avançar da idade. Os valores de PI_máx_ e PI_máx_ das meninas foram de 53,49±11,07 e 61,80±12,51cmH_2_O, respectivamente; e os meninos de 59,96±13,66 e 69,68±15,72 cmH_2_O, respectivamente.

**Conclusões:**

Foram desenvolvidas curvas e percentis normais para os valores de PI_máx_ e PI_máx_ de escolares saudáveis o que poderia auxiliar no acompanhamento clínico e terapêutico de diferentes populações pediátricas.

## INTRODUCTION

Respiratory muscle strength (RMS) normative values are essential to diseases and healthy individuals. For that, in diseases, maximal inspiratory pressure (MIP) and maximal expiratory pressure (MEP) are used to diagnose, indicate prognosis, and determine treatment. In health conditions, specifically for kids, assessing the RMS helps monitor growth and development.^1,2^

Usually, RMS studies in pediatrics have indicated adjusted models proposals,^3,4,5,6,7,8,9^ which allow only to explain a small part of the variability of the dependent variables; these variables are listed for the equations composition, elaborated to predict values of both MIP and MEP.

In general, the models use anthropometric factors such as age, weight, and height as predictor variables and have a very low adjusted coefficient of determination, around 30%.^4,8^ It is known that only two studies presented coefficients that exceeded 50%.^5,6^ These pieces of evidence indicate little accuracy in the proposed equations and suggest that the use of certain equations is more appropriate if restricted to the populations in which they were developed. This is because the proposal of these models to “predict” the average expected value is fragile because the “forecast error” factor is large.

Only estimating or predicting a child’s mean of MIP and MEP by predicted equations and normality values has not shown clinical significance, since it does not consider the variability of the measure for individuals with similar characteristics. This happens because not all individuals in the same community or region present MIP and MEP values equal to the average. The regional variability is widely discussed,^8,10,11,12,13^ but the variability of the measure itself is not. In this sense, analyzing whether or not the RMS measure verified in a patient by means of MIP and MEP values is adequate to the variability observed in their age and gender group seems to be substantial, since new proposals of equations for RMS parameters do not seem to meet the current clinical need.

A more elaborate methodological and data analysis design is necessary, such as developing a normal distribution and its percentiles. Thus, the objective of this study was to present standard references for MIP and MEP values of healthy schoolchildren, according to gender and ages between 7 and 10 years.

## METHOD

The standard reference was developed with data from a reference values study of maximum respiratory pressures of schoolchildren,^8^ approved by the Ethics and Research Committee on Human Beings of the State University of Santa Catarina (CAAE 01821712.6.0000.0118/Opinion 63455). In this study, healthy, collaborative, and eutrophic students, aged between 7 and 10 years, from three public schools in Florianópolis, belonging to a percentile of body mass index (BMI) >3 and <85 classified according to the Brazilian Ministry of Health,^14^ and capable of understanding and adequately performing all evaluation procedures were involved. The evaluations took place in the morning, with the schools’ permission and parents and child’s consent. The same examiner conducted the assessment of weight (precision 0.1kg) and height (precision 0.5cm), using a weight scale with an attached stadiometer (Welmy, 200/50g). The child remained barefoot, with an erect and aligned body, with minimal clothing to obtain these measures. The schools in the city were invited to participate in the study, and only three accepted the invitation, i.e., two private schools and one public, elementary, and high school, with approximately 500 students from each school. The three institutions are located in Greater Florianópolis and correspond to middle-class students On the occasion, ethical terms were sent to those responsible for each of the students, the Health Questionnaire the International Study of Asthma and Allergies Questionnaire (ISSAC), as well as the guidelines and objectives of the research. All students who returned with a signed parent’s consent and completed ISAAC and health questionnaire were evaluated. It was considered for inclusion in the statistical analysis only the RMS data of the students considered healthy.

The children’s health status was controlled by applying a health questionnaire developed by the researchers to control the absence of diagnosis or history of cardiorespiratory, musculoskeletal, rheumatic, neurological diseases, and auditory or visual deficits. The parents answered the questionnaire, and it had questions related to the practice of physical activity, home context, the use of medications, current or past illnesses, and history of hospitalizations. A questionnaire regarding respiratory symptoms, the ISAAC asthma module, was also applied, with questions related to the symptomatology of the past 12 months, with a total score of 14 points.^15^ Schoolchildren aged 7–9 years who presented scores equal to or higher than 5 points or schoolchildren aged 10 years with scores equal to or higher than 6 points due to the risk of asthma^16^ were not included in the study. Also, there were excluded children with acute illness at the time of data collection and those whose questionnaires were involved in the research had answers of questionable content. Children with forced expiratory volume in 1 second (FEV_1_) less than 80% of predicted by Polgar and Promadhat^17^ were also not part of the sample. FEV_1_ was obtained through a digital monitor (PIKo-1, nSpire Health, USA), following the American Thoracic Society (ATS) guidelines and performance criteria.^18^ The highest value of three measurements were recorded, with a 30-second interval between them, and two that did not differ by more than 0.15L in a maximum of five maneuvers.

Subsequently, maximum respiratory pressures were obtained through a digital manovacuometer with a one-way valve MVD300 (G-MED, Brazil) accuracy 0–300cmH_2_O, resolution 1cmH_2_O, and error of 1.8cmH_2_O. The measurements were performed by a single examiner and respecting the standards and criteria of the ATS.^19^ MEP was measured from an inspiration close to total lung capacity, followed by maximal expiration. The child expired up to near the residual volume and then carried out maximum inspiration in the sitting position to obtain the MIP. A minimum of three and a maximum of seven maneuvers were conducted for each of the measures of MIP and MEP. The measurements were considered satisfactory when the maximum value of three acceptable maneuvers (without leaks and lasting for at least 2 seconds) and reproducible varied less than 20% between them; the largest measure was recorded. There was an interval between 30 and 40 seconds between each maneuver of each measurement. Between the measurement of MIP and MEP, an interval of 3 minutes was guaranteed to avoid tiredness during an assessment. Only one evaluator conducted data collection; all evaluations of each subject were performed on the same day.

The MIP or MEP data obtained in the sample of healthy schoolchildren gave rise to standard references. The Lambda-Mu-Sigma (LMS) method^20,21,22^ was used to construct the curves. The software LMS chartmaker version 2.4 (Copyright 1997–2008; Medical Research Council, UK) was used to determine the L, M, and S parameters and the 3, 10, 25, 50, 75, 90, and 97 percentiles of the MIP and MEP. The z-score of any MIP or MEP value observed in children aged from 7 to 10 years from the L, M and S parameters was determined using the following formulas:^20^

$$\begin{array}{l} z=\frac{{\left(y/M\right)}^{L}-1}{L\times S}\;\;\text{if}\;\text{L\#0}\\ z=\frac{Log\left(y/M\right)}{S}\;\;\text{if}\;\text{L=0} \end{array}$$

## RESULTS

**Figure 1.**
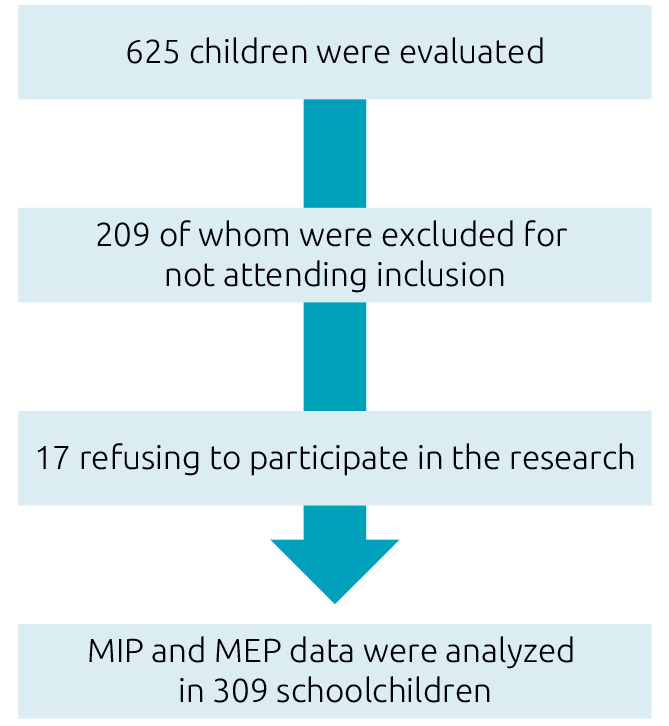
Flowchart of studied infants.

MIP: maximal inspiratory pressure; MEP: maximal expiratory pressure.

A total of 625 children were evaluated, and 209 of them were excluded for not meeting the inclusion criteria (Figure 1) and 17 refused to participate in the research. MIP and MEP data have been analyzed in 399 schoolchildren, of whom 101 were 7 years old (51 boys), 102 were 8 years old (50 boys), 101 were 9 years old (51 boys), and 95 were 10 years old (46 boys) (Table 1). All the students in the sample had adequate lung function and BMI.

**Table 1.** Anthropometric and ventilatory variables according to gender and age.

	Male (n=198)				Female (n=201)			
	7 (n=51)	8 (n=50)	9 (n=51)	10 (n=46)	7 (n=50)	8 (n=52)	9 (n=50)	10 (n=49)
Weight, kg	25.2±3.6	27.1±4.1	30.9±4.5	33.8±4.5	24.2±3.2	28.7±4.3	30.5±4.9	34.0±5.5
Height, m	1.3±0.1	1.3±0.1	1.4±0.1	1.4±0.1	1.3±0.1	1.3±0.1	1.4±0.1	1.4±0.1
BMI, kg/m^2^	15.8±1.1	15.6±1.3	16.6±1.4	16.3±1.4	15.4±1.2	16.6±1.3	16.4±1.5	16.8±1.5
FEV_1_L	1.4±0.2	1.7±0.2	1.8±0.2	2.1±0.3	1.4±0.2	1.6±0.2	1.8±0.3	2.0±0.3
FEV_1_%	93.1±7.1	95.9±6.8	94.7±6.1	93.8±7.3	93.0±9.6	94.0±5.5	93.0±6.4	94.2±6.4
MIP	54.6±11.3	56.8±11.1	61.4±10.7	64.3±13.2	48.6±8.1	52.3±10.2	53.7±10.2	56.2±9.1
MEP	62.8±12.7	65.3±11.6	75.3±15.7	74.4±14.7	54.5±9.8	60±10.5	63.2±12.3	66.2±9.4

All variables are expressed in mean±standard deviation. FEV_1_L: forced expiratory volume in the first second in liters; FEV_1_%: percentage of forced expiratory volume in the first second in relation to predicted; MIP: maximal inspiratory pressure, MEP: maximal expiratory pressure; SD: standard deviation; Min: minimum value; Max: maximum value.

**Figure 2.**
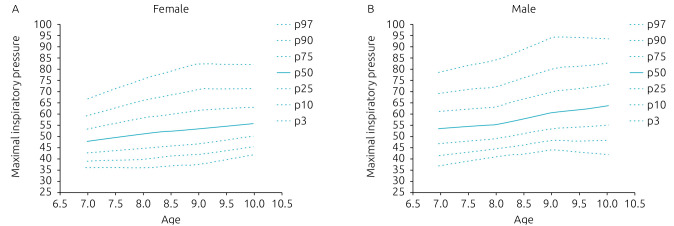
Normal distributions, according to each percentile, of the maximal inspiratory pressures for females (A) and males (B).

**Figure 3.**
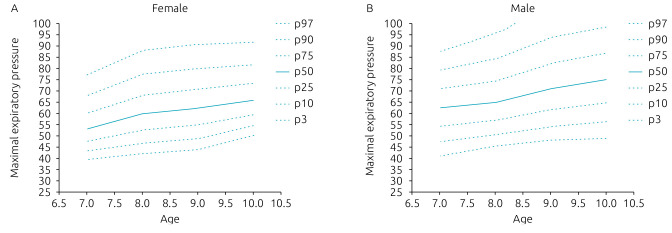
Normal distributions, according to each percentile, of the maximal expiratory pressures for females (A) and males (B).

Tables 2 and 3 show the percentiles (P3, P10, P25, P50, P75, P90, and P97) of MEP and MIP, respectively, of girls and boys, between 7 and 10 years old. Normal data distribution of _+_MIP represents the percentiles behavior, according to the age of both genders, which are presented in Figure 2. Figure 3 shows the normal distribution for the MEP curves. There was an increase in the values of the maximum respiratory pressures with the progression of age.

**Table 2.** Maximal expiratory pressure percentiles in 7- to 10-year-old boys and girls.

Age, years	Female										Male									
	L	M	S	P3	P10	P25	P50	P75	P90	P97	L	M	S	P3	P10	P25	P50	P75	P90	P97
7	-0.6	53.1	0.1	39.5	43.2	47.5	53.2	60.1	67.8	77.2	0.5	62.3	0.1	41.0	47.3	54.2	62.4	71.0	79.2	87.6
8	-0.2	59.8	0.1	42.0	46.8	52.5	59.9	68.5	77.6	88.1	-0.1	64.8	0.1	45.3	50.6	56.9	64.9	74.3	84.2	95.6
9	-0.2	62.3	0.1	44.0	49.0	54.9	62.4	71.2	80.4	91.0	-0.1	71.0	0.2	48.0	54.3	61.5	71.0	82.2	93.9	107.5
10	-0.6	66.0	0.1	50.3	54.6	59.6	66.1	73.8	82.1	91.9	0.2	75.1	0.2	48.9	56.3	64.7	75.1	86.9	98.6	111.5

L: skewed value; M: mean; S: coefficient of variation; P3: percentile 3; P10: percentile 10; P: percentile 25; P50: percentile 50; P75: percentile 75; P90: percentile 90; P97: percentile 97.

**Table 3.** Maximal inspiratory pressure percentiles in 7- to 10-year-old boys and girls.

Age, years	Female										Male									
	L	M	S	P3	P10	P25	P50	P75	P90	P97	L	M	S	P3	P10	P25	P50	P75	P90	P97
7	-0.6	47.6	0.1	36.1	39.2	42.8	47.6	53.3	59.5	66.8	-0.1	53.2	0.2	36.9	41.3	46.5	53.2	61.1	69.3	78.7
8	-0.4	50.9	0.1	36.2	40.2	44.8	51.0	58.3	66.3	75.8	-0.8	55.3	0.1	40.6	44.4	49.1	55.3	63.3	72.4	84.2
9	-0.5	53.4	0.2	37.8	41.9	46.8	53.4	61.7	71.0	82.5	-0.7	60.8	0.1	43.8	48.2	53.5	60.8	70.1	80.7	94.3
10	-0.9	55.7	0.1	42.0	45.6	49.9	55.7	63.1	71.5	82.3	0.1	63.6	0.2	42.0	48.1	55.0	63.7	73.4	83.2	93.9

L: skewed value; M: mean; S: coefficient of variation; P3: percentile 3; P10: percentile 10; P: percentile 25; P50: percentile 50; P75: percentile 75; P90: percentile 90; P97: percentile 97.

## DISCUSSION

This study determined the extreme percentiles (3rd and 97th) for MIP and MEP values of healthy children of three schools of Florianópolis. Reference values for RMS of Brazilian children were proposed in several country regions;^3–9^ although there is wide variability, these constitute clinical parameters of MIP and MEP. To date, percentiles and curves of normal distribution of RMS for Brazilian children have not yet been proposed.

When plotting curves and percentiles, at least 100 cases are required for each age group for percentile accuracy. However, the curves presented here were developed for each gender, totaling 50 cases in each curve, which can be considered a limitation. In clinical practice, having access to reference values that express the observed variability in the population is essential for health professionals who follow the growth and development of the pediatric age group. Additionally, identifying whether a child has RMS above or below average or even 25% above average does not translate to the health professional, a physician, or physiotherapist who will propose a muscle training program; the actual clinical condition; and potential evolution of an individual. Nevertheless, highlighting that an RMS-related parameter, such as the MIP of a child, is less than the 5th percentile, which means that 5% or less of the healthy children have MIP lower than hers, is consistent information. Although the sample is characterized by the origin of public schools and, consequently, low income is possible, the majority corresponded to values of MIP and MEP compatible with data established in the literature. That said, social income does not seem to have compromised the examination’s understanding, and RMS was measured indirectly by manovacuometry.

Recently, Hulzebos et al.^23^ established distribution curves for healthy Dutch children and young people, aged from 8 to 19 years, based on reference values of 117 boys and 134 girls. The adjusted coefficient of determination values was close to 50%; however, when subdividing the sample into ages, the authors determined percentiles based on subgroups composed of an average of 20 children. Furthermore, unlike the present study, which included a narrower age group (ages 7–10 years), it was restricted to pediatrics and included 50 students for each age group.

However, on the differences of the age group and average values of the RMS, it is observed that when comparing with other populations, our MIP and MEP averages are lower.^5,23^ In addition to the different age groups, in the present study, we used the specific reference equation for the population.^4^ Furthermore, the anthropometric differences between children of the same age group are considerable.^5^ These findings reinforce the guidance of the ATS to develop reference values and specific normality curves for each population.

Attention exclusively to the behavior of the pediatric age group becomes relevant since the study of RMS in this population has been the focus of recent investigations,^24,25,26^ mainly since muscle strength is impaired by typical diseases and related to childhood. There are neuromuscular diseases, which evolve with progressive weakness of the intercostal and diaphragmatic muscles and the abdominal muscles. This decrease in strength determines the ineffectiveness of cough, a deficit in the clearance of airway secretions, dysfunction of respiratory mechanics, and postural changes, which usually results in respiratory failure.^27^ Therefore, the application of specific protocols for RMS training in this population, although still controversial,^26^ seems to improve not only the measured values of MIP and MEP itself but also the peak of cough flow, fundamental for maintaining the integrity of the respiratory system.^28^ In asthma, respiratory muscle training appears to increase MIP and MEP, as well as expiratory peak flow, which suggests a reduction in airway obstruction.^29^ In children with cystic fibrosis (CF), RMS changes are still not well characterized. MIP and MEP values may be preserved, attributed to a possible “training” effect due to increased respiratory work resulting from the disease progression^24^ or decreased due to pulmonary hyperinflation and malnutrition.^24^ However, RMS in this population can be altered with physical training,^8^ and a study has identified higher MIP and MEP values in patients with CF who exercise regularly compared to those who do not.^8^ However, the application of specific training is still controversial since there is still insufficient evidence on the benefits of this intervention. It is relevant to emphasize that the proper prescription of respiratory muscle training depends directly on the proper evaluation and applicability of the curves.^26^

In turn, in healthy children, RMS has been related to anthropometric measures and physical activity levels.^30,31,32^ BMI, for example, is strongly related to RMS, probably due to the restriction caused by adipose tissue that causes respiratory work overload.^30–32^ In addition to these factors, the physical activity level of healthy children is also related to RMS since higher values of MIP and MEP are related to physical activity practice.^28^ Thus, physical training programs, even nonspecific ones, can improve RMS parameters.

In this context, the applicability of the normal distributions extrapolates disease conditions. However, this seems to be indicated for the prevention and control of the entire infantile population, since RMS can be altered due to chronic respiratory and neurodegenerative diseases and postural and childhood obesity. Therefore, the evaluation, classification, and follow-up of representative parameters of RMS, such as MIP and MEP values, through an instrument of easy application and interpretation, such as the curves presented here, can guide the control of diseases progression and therapeutic interventions, assist the prescription of more specific training, and facilitate the detection of conditions that may influence the quality of life of a child.

One limitation of the current research is that the curves were developed based on data from schoolchildren from only one city in Brazil, which can compromise the external validity of the research and the consequent generalization of the results to the behavior of the RMS for all students in the country. However, considering that, in the Southern region, Florianópolis has great ethnic diversity, whose inhabitants have had influences from Portuguese, German, Italian, Japanese, Austrian, and Polish immigrants, it is a city that reflects the character of the country’s miscegenation. In addition, it is one of the capitals that receive the most migrants from different states and Haitians due to the good quality of life it offers, which also contributes to a more eclectic profile of schoolchildren.

The standard reference and percentiles were presented for healthy schoolchildren’s MIP and MEP values developed according to gender and ages from 7 to 10 years. In addition, the extreme percentiles (3rd and 97th) were determined, and a specific graph was elaborated for each group, which could contribute to the clinical and therapeutic monitoring of different pediatric populations.
